# Ultra-Low Dosage Regimen of Rituximab in Autoimmune Blistering Skin Conditions

**DOI:** 10.3389/fimmu.2018.00810

**Published:** 2018-04-18

**Authors:** Mauro Alaibac

**Affiliations:** Unit of Dermatology, Department of Medicine, University of Padua, Padua, Italy

**Keywords:** rituximab, autoimmune blistering conditions, skin, pemphigus, B-cells

Rituximab is a chimeric human-mouse monoclonal anti-CD20 antibody initially developed to treat B cell lymphoproliferative disorders (1). It is now increasingly being used for the treatment of B cell-mediated skin autoimmune disorders, including autoimmune blistering skin diseases (2, 3). Rituximab targets all CD20 expressing B-cells, including precursor B cells and mature and memory B cells, whereas it does not interact with CD20-negative early pre-B cells and terminally differentiated plasma cells. Consequently, it completely depletes peripheral memory B cells, but it does not affect preexisting titers of serum antibody produced by long-lived antibody-secreting plasma cells (4). Rituximab mainly acts through antibody-dependent cell-mediated cytotoxicity, although alternative mechanisms may also be involved in complement-dependent cytotoxicity and in direct effect of the drug leading to apoptosis (5). Peripheral B cell depletion by rituximab treatment is usually observed for 6–9 months. After 1 year, many patients show complete mature B cell recovery, whereas the population of circulating memory B cells may be slow to recover after treatment (6). On the other hand, it is plausible that rituximab treatment does not completely eliminate memory B-cells which could be responsible for subsequent disease relapse.

Autoimmune blistering skin diseases are a group of heterogeneous skin conditions characterized by the presence of serum autoantibodies targeting desmosomal structural proteins (pemphigus group) or the hemidesmosomal anchoring complex (pemphigoides group) (7, 8). These disorders are generally treated with systemic corticosteroids which are often combined with other immunosuppressive and/or immunomodulatory approaches, notably azathioprine, mycophenolate mofetil, dapsone, tetracyclines, plasmapheresis, immunoadsorption, and high-dose intravenous immunoglobulins (9). These treatments may not be effective for either induction or maintenance of remission or, alternatively, need to be discontinued because of unmanageable adverse effects. B-cells play a central role in the pathogenesis of autoimmune blistering disorders and rituximab, which selectively targets B cells, has proven to be an effective and safe therapeutic agent in patients with autoimmune blistering skin disorders refractory to conventional treatments (9). To this regard, the recent published randomized controlled trial of rituximab and short-course prednisone versus standard-dose prednisone in new-onset pemphigus has clearly demonstrated that rituximab with short-course prednisone was a more effective treatment than the high-dose prednisone regimen used in this trial, which has been the mainstay of therapy for pemphigus over the years (10). Furthermore, rituximab associated with short-course prednisone showed lower rates of grade 3 or 4 adverse events compared with standard-dose prednisone (10). These results indicate that rituximab associated with short-course prednisone should be considered the first-line therapy for new-onset pemphigus.

The optimal dosing of rituximab in autoimmune blistering skin conditions is currently poorly defined. Initially, rituximab was used for the treatment of pemphigus following the lymphoma dosing regimen (375 mg/m^2^ weekly for 4 weeks) (11). However, given the fact that B-cell burden in autoimmune blistering skin diseases is much lower than that in lymphoproliferative disorders, several studies have investigated lower dosage. Horwath et al. treated patients with pemphigus with a single course of two infusions of rituximab (500 mg each) at an interval of 2 weeks with satisfactory responses and relapses generally occurring at the end of the second year (12). Several low-dose protocols for the treatment of pemphigus have been reviewed in a recent meta-analysis, such as the rheumatoid arthritis protocol (2 × 1,000 mg doses at a 2-week interval) and the standard low-dose rituximab (2 × 500 mg doses at a 2-week interval) with the concomitant use of immunoadsorption or high-dose intravenous immunoglobulins (13). Moreover, a very recent investigation has shown impressive results in healthy volunteers treated with ultra-low dosage of rituximab. In this study, the authors demonstrated that <1% of the conventional rituximab doses induced a nearly complete depletion of circulating B lymphocytes (1 mg/m^2^ rituximab depleted 97% of all B-cells and doses of 0.3 mg and 0.1 mg/m^2^ depleted, respectively 75 and 66% of circulating B-lymphocytes) (14). After 4 weeks from the infusion of rituximab circulating B-cells returned to approximately 60% of normal levels in the 1 mg/m^2^ dose group, whereas recovery was completed by 9 months after infusion in the 1 mg/m^2^ dose group and about 1 month after infusion in the 0.3 mg and 0.1 mg/m^2^ dose groups. From the data of the study, the authors extrapolated that 100 mg rituximab may be sufficient to induce a depletion of B-cells for 3 months and, consequently, two doses of 100 mg every 3 months could deplete the B-cell population for 6 months. Thus, alternative ultra-low dosing schedules for rituximab could be proposed for antibody-dependent autoimmune diseases, including autoimmune blistering skin disorders, as they may provide a considerable improvement of the cost effectiveness. Moreover, well-designed clinical trials are warranted to determine the efficacy of ultra-low dosage rituximab and to assess whether there are other potential advantages over conventional low-dose protocols, in particular lower risk for infection and lower risk for infusion reaction.

## Author Contributions

MA conceived and wrote the manuscript.

## Conflict of Interest Statement

The author declares that the research was conducted in the absence of any commercial or financial relationships that could be construed as a potential conflict of interest. The reviewer MS and handling Editor declared their shared affiliation.
